# *In Silico* Study to Develop a Lectin-Like Protein from Mushroom *Agaricus bisporus* for Pharmaceutical Application

**DOI:** 10.3797/scipharm.ISP.2015.11

**Published:** 2016-02-14

**Authors:** Wangsa Tirta Ismaya, Yunita, Sophi Damayanti, Caroline Wijaya, Raymond R. Tjandrawinata, Debbie Sofie Retnoningrum, Heni Rachmawati

**Affiliations:** 1 Dexa Laboratories of Biomolecular Sciences, Industri Selatan V, Blok PP No. 7, Kawasan Industri, Jababeka II, Cikarang 17550, Indonesia; 2 Research group of Pharmaceutics, School of Pharmacy, Institut Teknologi Bandung, Jl. Ganesa 10, Bandung 40132, Indonesia; 3 Research group of Pharmacochemistry, School of Pharmacy, Institut Teknologi Bandung, Jl. Ganesa 10, Bandung 40132, Indonesia; 4 Research group of Biotechnology, School of Pharmacy, Institut Teknologi Bandung, Jl. Ganesa 10, Bandung 40132, Indonesia

**Keywords:** *In silico* protein study, Lectin-like protein, Light subunit mushroom tyrosinase, Molecular docking, Novel pharmaceutical protein, Oral delivery

## Abstract

A lectin-like protein of unknown function designated as LSMT was recently discovered in the edible mushroom *Agaricus bisporus*. The protein shares high structural similarity to HA-33 from *Clostridium botulinum* (HA33) and Ricin-B-like lectin from the mushroom *Clitocybe nebularis* (CNL), which have been developed as drug carrier and anti-cancer, respectively. These homologous proteins display the ability to penetrate the intestinal epithelial cell monolayer, and are beneficial for oral administration. As the characteristics of LSMT are unknown, a structural study *in silico* was performed to assess its potential pharmaceutical application. The study suggested potential binding to target ligands such as HA-33 and CNL although the nature, specificity, capacity, mode, and strength may differ. Further molecular docking experiments suggest that interactions between the LSMT and tested ligands may take place. This finding indicates the possible use of the LSMT protein, initiating new research on its use for pharmaceutical purposes.

## Introduction

Proteins of Lectin family have been assessed for application as drug carrier because of their capability to recognize and bind sugar moieties. Depending on the type of sugar, lectin can be employed to deliver drug or therapeutic protein to different target, i.e. mucosa (galactose [GAL], *N*-acetyl-galactosamine [NGA], and *N*-acetyl-glucosamine [NAG]) or gastrointestinal track (NAG, mannose [MAN], *N*-acetyl-sialic acid [ASA], glucose [GLU], and GAL) [2]. This capability promotes their use in drug delivery via oral route, the most favorite option of drug administration [41]. This route is often hampered by the ability of the drugs to pass through the epithelial cells of intestine. On the other hands, peptides or proteins based drugs and carrier usually not survive upon exposure to gastrointestinal track conditions, such as acidic and presence of proteases [35]. Whilst designing a delayed- or timed-release of the drugs has solved the latter issue, liposome and cell-penetrating peptide (CPP) have been developed to overcome issues with delivery of the drugs across the epithelial cell. However, the use of liposome is limited by the size of the drug can be carried and by the hydrophobic nature of the carrier [35], while the use of CPP is hampered by unclear mode of internalization and its potential immunogenicity [35]. Therefore, employing proteins capable of penetrating the epithelial cell of intestine is increasingly popular. In particular for therapeutic proteins, the carrier protein can be produced as a fusion with the therapeutic protein load. The fusion protein is equipped with a cleavage site to ensure the release of the load. Preparation of such carrier would benefit the patient from belonephobia, sterility upon use, air boluses, even inexperience use of injection tools upon self-administration, and from ease of administration.

Recently, a protein of unknown function is discovered in the mushroom *Agaricus bisporus* upon elucidation of the structure of PPO3 (PDB ID 2y9w and 2y9x), one of mushroom *A. bisporus* tyrosinase isoforms [17]. The protein has consistently been found and addressed as the light subunit of mushroom tyrosinase (LSMT), which its encoding gene (*orf239342*) was found to cluster in the same chromosome with the genes coding for PPO [49]. The protein was not identified as the mushroom lectin [10] but shares a typical β-trefoil fold of lectin. Unfortunately, apart from this structural information, practically nothing is known about its characteristics. Most importantly, LSMT shares high structural homology with HA-33 from *Clostridium botulinum* (HA-33, PDB ID 3AH2, r.m.s.d 0.97 Å for 106 of 140 amino acid residues) and Ricin-B like lectin from mushroom *Clitocybe nebularis* (CNL, PDB ID 3NBE, r.m.s.d 1.18 Å for 90 of 140 amino acid residues), which both have been developed for pharmaceutical applications.

HA-33 is one of the non-toxic non-hemagglutinin components in the 16S progenitor toxin from *C. botulinum*. The protein is a 284 amino acid long and forms a β-trefoil configuration [1], which is a typical fold for proteins of lectin family. The protein is part of the Botulinum neurotoxin (BoNT) complex that assists the absorption of the toxin by intestinal epithelial cells [18]. The mechanism of HA-33 penetration into intestinal epithelial cell monolayer is still a puzzle [13]. HA-33 appears to bind epithelial cells and likely facilitates attachment of the toxin to the apical surface of the cells and receptor-mediated endocytosis [12]. The protein is usually in complex with HA-17, which altogether undergoes conformational change in the presence of sugars [37]. Importantly, this protein is remarkably resistant to proteases and acidic pH thereby survives the conditions of gastrointestinal tract [29]. Therefore, HA-33 has been developed as drug carrier [27].

Unlike HA-33, CNL is member of lectin family and isolated from mushroom [31]. Thus, CNL appears to be more related to LSMT than HA-33. CNL exhibits agglutinating activity, binds various type of sugars, and display immuno-stimulatory and anti-proliferative activity [31, 44]. The latter promotes CNL for use in therapeutic strategy in strengthening anti-tumor immune response. CNL has a Ricin-B like fold [33] and displays toxicity against insects, amoebozoa, and nematodes [3, 32]. Toxicity of CNL is originated the binding of sugar component of glycoprotein or glycolipid, facilitating internalization and intracellular transport of the protein [51]. The unique feature of CNL is recognition to *N*-*N*’-2-acetyllactosediamine (ALD), which is a glycan structure rarely found in mammal, restricted only to glycoprotein in hormone [46] and in human Jurkat leukemic cells thus is associated with malignancy [16, 24, 33]. Therefore, CNL is developed as an anti cancer agent. Lectin from mushroom *A. bisporus* has also been developed as an anti-cancer agent [23], for demonstrating inhibition to proliferation of epithelial cell of the colon.

During evaluation of their amino acid sequences, the carbohydrate-binding residues in HA-33 and CNL appeared to be not conserved in LSMT [27, 33], therefore binding to carbohydrate or attachment to cell wall was proposed to be unlikely [17]. However, the sugar binding residues of HA-33 are different to those of CNL despite sharing the same binding pocket with nearly identical architecture. Interestingly, this binding site architecture is also prevailed in LSMT, therefore opens an opportunity for evaluating its possible function and further possible pharmaceutical application.

In order to assess that possibility, the LSMT structure was subjected to *in silico* structural analysis to find possible interaction with other biomolecules. The study was initiated with defining region on the protein available for interaction with other biomolecules and to screen for biomolecule to test. Potential binding energy is calculated from validated molecular docking experiments, in which docking of ALD onto CNL was also performed as a comparison. Further, the structure of LSMT was also compared to that of CNL and HA-33 to gain further insight into possible interactions at molecular level. Based on docking of the tested biomolecules and structural assessment, possible pharmaceutical application was devised. Most importantly, this *in silico* study serves as the concept for our current investigation that provides experimental evidences, which will soon be published.

## Results and Discussion

Numerous amount of proteins in the database (for both amino acid or gene sequences) still have no assigned biological function even after their structures have been elucidated [26]. This includes LSMT, which its structure was discovered serendipitously during the elucidation of the structure of PPO3 [17]. This protein was initially speculated as the C-terminal fragment of the tyrosinase subunit, which its identity was also ambiguous prior to elucidation of its structure. Even today, information on LSMT *i.e*. its presence and nature in cellular compartment, characteristics, and even association to PPO3, is unknown. So far, our effort to isolate LSMT is fruitless whilst preparing its recombinant version would be hampered by validation of the gene product. Nevertheless, similarity of LSMT structure with its structural homolog allows prediction of its functionality [22], because protein structure is more conserved than amino acid [36] and dictates its functionality [14]. Therefore, performing *in silico* study appears as an appealing alternative to propose the function and potential use of LSMT.

### Search for potential binding site

**Fig. 1:**
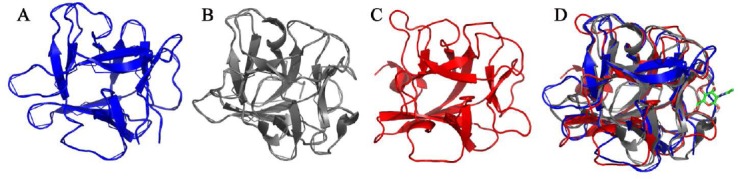
Structural comparison of LSMT (A), CNL (B), HA-33 (C). Predicted potential ligand molecules in the binding site region LSMT, ligand binding-sites of CNL and HA-33 are indicated by an NAG molecule (in stick) in the superimposed structures (D). Each structure is colored accordingly.

The program FTSite [4, 28] predicted the presence of two sufficiently large cavities for the binding of molecules onto the surface of LSMT. In the structure of PPO3-LSMT complex, the first site occupies the interface region between the two subunits while the second is located at the opposite site in the molecule. Interestingly, the second putative ligand-binding region in LSMT structure highly resembles the sugar-binding site in the structures of CNL and HA-33. This region is in vicinity of a long surface loop (fragment 29–34 in the amino acid sequence) that is missing in the structure of LSMT [43]. This surface loop would not interfere with ligand binding due to its high flexibility. Thus, the structural architecture perspective permits binding of ligands in that region. Most importantly, the secondary structures of CNL, HA-33, and LSMT in that region are similar thereby potential interactions could be predicted (Figure 1a–d).

The program 3DLigandSite [48] confirmed the prediction of the second ligand-binding site. Molecules predicted to suit for interactions were NAG, fucose (FUC) and NGA. Interestingly, they are among type of sugars decorating mucosa or gastrointestinal track targeted by lectins [2].

Amino acid residues responsible for binding of the sugars in lectins are previously reported to be absent in LSMT [17]. However, this study revealed that the predicted binding site for ligand in LSMT is located in similar region to that of CNL and HA-33. The architecture of the binding sites is also very similar. The result of this structural similarity updates the previous hypothesis, which is based on the output of comparison of amino acid sequence. Furthermore, although HA-33 and CNL display capability to penetrate intestinal epithelial cell monolayer, the residues responsible for binding of ligands are not shared. Thus in principle, LSMT could also perform such interactions. Finally, additional analysis using the program Findsite [52] supported the prediction on the binding of FUC and NAG to LSMT, as well as location of the binding site.

### Assessment of the potential binding site

Further assessment was carried out with molecular docking programs to evaluate whether the proposed interactions are not only structurally possible but also energically favorable. Upon initial molecular docking experiment employing Autodock4.2.5, negative binding energies were obtained from thermodynamics calculation when tested ligands were docked into the putative binding pocket of LSMT. This output indicates that interaction between tested ligands and LSMT may take place [5], although the binding appears to be weak. However, the calculated binding energy values obtained from Autodock4.2.5 were still quite varying, with deviation up to 25%. Therefore, molecular docking experiment was further carried out using Autodock Vina, focused on this putative binding region. The output of Autodock Vina was similar to that of Autodock4.2.5 but with better statistics (Table 1).

**Tab. 1:**
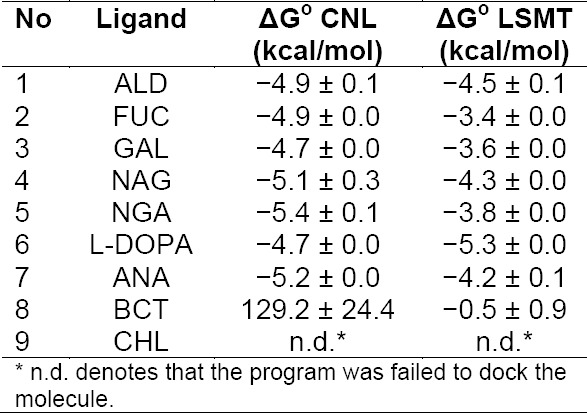
Prediction of interaction between LSMT and tested ligands from molecular docking with Autodock Vina. CNL was included to provide illustration of its interaction with the natural ligand partner.

As a comparison, docking was also done in parallel using the structure of CNL and its ligands, ALD and GAL. In the crystal structure of CNL, GAL and ALD adopts the same position, in which the pyranose ring of GAL overlaps perfectly with that of ALD [33]. Upon docking, both GAL and ALD docked quite well in the putative binding site of LSMT, adopting similar orientation as in CNL. Binding of FUC, NAG, and NGA was also predicted to involve their pyranose rings, indicating that sugars containing this structural feature might be suitable ligand for LSMT.

Control compounds of organic molecule containing ring structure were recruited to test for specificity of the binding. L-3,4-dihydroxyphenylalanine (L-DOPA) was chosen because of LSMT association with tyrosinase. This compound is well known as the substrate for the enzyme[40]. N-acetyl neuraminic acid (ANA) was the next to test because the molecule is a derivative of monosaccharide that has been reported to associate with lectin recognition to glycan [47]. The docking result suggested that both LSMT and CNL are able to bind these compounds, indicating for binding of any compounds with ring structure. To test this indication, β-carotene (BCT) was recruited. The testing output suggested that BCT was not suitable for docking onto both CNL and LSMT. Further testing was carried out with CHL because the molecule was predicted to bind LSMT, in the preliminary site search. The molecular docking program was failed to dock the molecule. These latter results suggest that LSMT (and CNL) are able to bind molecule with ring structure but not randomly. Furthermore, the number of possible conformation adopted by the molecule upon docking indicated that all molecules with good binding energy have nine possible conformations whilst BCT has only one.

### Reconstruction of ligand binding

Details of interaction between LSMT and the ligand were further evaluated structurally through inspection of amino acid residues reside in vicinity of the ligand. Residues with orientation supporting for interaction within a distance of up to 3.5 Å were considered. In CNL, Oδ of Asp20, Nδ of Asn38, and Nδ of Asn46 facilitate interaction with the pyranose ring of ALD whilst interactions with the peptide bone of Thr22-Gly23 and Oγ of Ser24 (CNL amino acid sequence numbering) stabilize the binding [33]. Interestingly, amino acid residues responsible for interaction in CNL are equally present in LSMT. Binding residues proposed for interaction in LSMT are Asn24, Ser26, Thr37, Leu38, Asp42, and Ser44. Those residues are located in proximity, indicating that they actually reside in the same binding pocket. This observation also supports the validation of the docking, which shows minimum variation in the calculated binding energy.

**Fig. 2:**
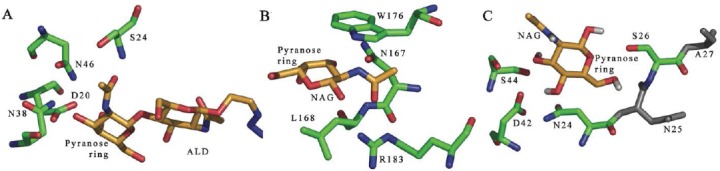
Interactions between ligand and protein in CNL (a), HA-33 (b) and the reconstruction in LSMT (c). Depicted amino acids are numbered according to their respective position in the amino acid sequence of each protein.

ALD is bound to CNL through hydrogen bonds with residues Asp20, Ser24, Asn38, and Asn46 (CNL amino acid sequence numbering) (Figure 2a). Similar interactions may take place in LSMT, involving Asn24, Asp42, and Ser44 (LSMT amino acid sequence numbering) (Figure 2c). Position and orientation of these residues in LSMT and CNL are equal, implying that interactions in CNL can mostly be reconstructed in LSMT.

Interaction between the ligand and protein in HA-33 is rather different. The NGA molecule forms hydrogen bonds with Asn167 and Arg183, and stabilized by hydrophobic interaction involving Leu168 and Trp176 (HA-33 amino acid sequence numbering) (Figure 2b) [27]. Asn167 of HA-33 interacts with the hydroxyl groups of the sugar through hydrogen bonding. Positions of Asn167 and Leu168 residues in HA-33 are occupied by Ser26 and Ala27 (LSMT amino acid sequence numbering), in which Ser26 was predicted to be involved in the interaction (Figure 2c). Asn167 and Leu168 reside in the beginning of surface loop that equals to the missing surface loop in LSMT. This missing surface loop is long and flexible, and hosts Asn30 and Leu31, which if in existence may have similar function to Asn167-Leu168 of HA-33. This analysis suggests that interaction of HA-33 with its substrate is less replicated in LSMT.

The output of molecular docking suggests that ligand binding in LSMT may resemble more of CNL than HA-33. However, HA-33 was not yet to be excluded from structural homology because of similarity in its binding pocket architecture to LSMT. The structure of HA-33 may still be useful in reconstructing the potential binding pocket of LSMT.

**Fig. 3:**
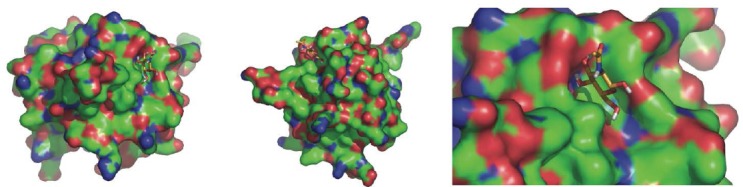
Predicted docking of NAG on the surface of LSMT; presented at different angle and zoomed into the binding site.

The size of the putative binding pocket of LSMT is about 4Å deep, 14Å long, and 6Å wide, sufficiently large to accommodate binding of a molecule. Major interactions appear to occur at one side of the groove as indicated by the NAG molecule docked (Figure 3).

Structural comparison suggests that the binding of sugars in the binding pocket of LSMT is plausible because the amino acid residues that facilitate interactions are actually available. Molecular docking study of LSMT and structural comparison to CNL and HA-33 indicates that the molecular detail of interactions between LSMT and potential ligands resembles more of that of CNL than HA-33, involving Asn24, Ser26, Thr37, Leu38, Asp42, and Ser44 (Figure 2c). Differences between CNL, HA-33 and LSMT may indicate that LSMT is capable of binding different type of sugar, has different affinity towards sugar, different strength, or different fashion of binding.

### Potential immunogenicity

HA-33, CNL, and LSMT are proteins of foreign origin that potentially is recognize by our defense system as an antigen. Introduction of bacterial recombinant HA-33 is found to evoke high titer of antibody in mice [42]. CNL demonstrates immune stimulatory activity in vitro, as shown by activation and maturation of DC cells [44]. However, although CNL appears to stimulate innate immune response, it is not yet indicated to provoke adaptive immune response, therefore possibility of its clearance by the antibody is not yet firmly established. Furthermore, no experiments have been reported to show that both proteins are indeed immunogenic in animal models or human.

*In silico* experiments were conducted to predict possibilities of immunogenicity of LSMT. Using as low as 60% possibility as the cut off, prediction with the program Ellipro revealed the presence of four linear and three discontinuous B cell epitopes in LSMT, respectively [34]. That prediction was confirmed by other prediction tools such as BCPreds [8], AAP [6], ABCpred [39], Bcepred [38], Discotope [20] and Bepipred [21]. The final consensus is presented in table 2. The length of predicted epitopes are between 7–8 amino acid residues, which is actually rather short. Also, of the four peptides presented, only two peptides are scored above a confident level of 75%. Prediction with Ellipro is based on the protein structure while other programs are mostly based on amino acid sequence. Therefore, the output of Ellipro is adopted as the primary result. The prediction output was not really suggesting immunogenicity of the protein because the recognizing peptide is short and the level of confident is below 85%. Thus, the prediction suggests that LSMT could be recognized as an antigen but the chance is low. Nevertheless, its recognition by human antibody is still to be evaluated. LSMT has so far not been reported as toxin or toxin component. The protein is present in the edible button mushroom fruiting bodies, which are part of our dietary.

**Tab. 2:**
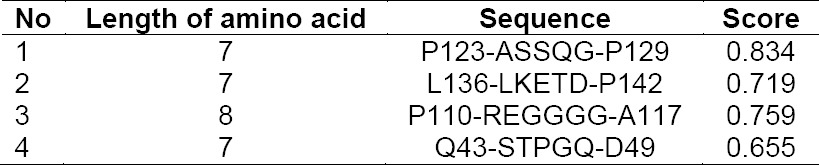
Consensus of predicted regions for antibody recognition. The score refers to the output of Ellipro.

Our *in silico* study showed that LSMT is capable of recognizing sugars that are natural ligands of its lectin and lectin-like protein homologs. Thus, the protein can potentially be developed for pharmaceutical purposes, as its homologs have been. Further, in the absence of characteristics and biochemical information on the protein, the study may provide insights to the functioning of the protein in nature, derived from type of molecules that are capable to dock in its binding site. In addition, we have recently performed study *ex vivo* and *in vitro* using mice that provides experimental evidence for the hypothesis presented. The results are being reviewed and will soon be published.

## Experimental

### Protein models

The structure of LSMT was extracted from the structure of mushroom tyrosinase quaternary complex at 2.3 Å resolution (PDB ID 2y9w), removing all notations for molecules except for that of chain C. The protein structure was checked with the program COOT to ensure no structural defects from manual modification [9]. Search for protein homolog was initiated with amino acid sequence similarity search using the online program BLAST [19]. The search was continued using the online program DALI for proteins of similar structural fold and architecture [15]. The structures of LSMT closest homolog (CNL: PDB ID 3NBE, and HA-33: PDB ID 3AH2) were downloaded from the PDB database. The structure of CNL was modified by means of removing notation for molecules except for the chain A and its ligand. The structure of HA-33 was truncated to contain only the C terminal domain (residues 147–286) of the chain B and the ligand.

### Search for binding site and potential ligand

In search for possible binding sites and ligands, LSMT structure was submitted to the program FTSite [4, 28]. The output of analysis was visualized online with the viewer embedded in the program and then compared to 2y9w and to LSMT model. The two latter models were reoriented according to the output of FTSite. To prevent speculation, result of the FTSite analysis was employed only to indicate regions possible to facilitate interaction with different biomolecule.

Further search was done by the program 3DLigandSite [48]. A total of 15 ligands from 10 different structures were recommended, of which were GAL, NAG, NGA, chlorophyll (CHL), and FUC. CHL were excluded because of its lack of biological relevance to LSMT and pharmaceutical interest. Six of eight amino acids were recorded as potential residues for interactions: Asn24, Ser26, Asn30, Ile40, Ser44, and Thr45. Additional analysis was done using the program Findsite-comb [52]. From the initial 82 structure templates and 171 bound ligands, only ten templates and ligands survived the first screening of template filtering. Most importantly, five possible binding pockets were found, of which only one was regarded as highly potential. This binding pocket was recorded and considered in evaluating structural model of LSMT and of its homologs.

Based on those results, further structural evaluation was conducted. First, structures of CNL and HA-33 were independently superimposed onto that of LSMT using the program LSQKAB from the CCP4 program suite [50]. ALD and NAG (natural ligands of CNL and HA-33, respectively) were extracted from their crystal structures and imposed onto the structure of LSMT to find correlation between the three structures. The position of ALD and NAG were also adopted for initial positioning of the ligands for molecular docking. Furthermore, location of the grid during molecular modeling was referred to the region in which ALD and NAG are bound.

### Preparation of ligands

Ligands selected for molecular docking were based on sugars predicted from initial structural homolog and ligands search results. The ligands used in molecular docking were built using molecular builder on GaussView 5.0 and optimized to the lower energy configuration using Gaussian09w on 6-31G basis set and Becke three-parameter Lee-Yang-Parr (B3LYP) hybrid Density Functional Theory (DFT) [7, 11]. The ligands were prepared by adding all hydrogen atoms and the partial atomic charges that was calculated using Gasteiger charge and merged all the non-polar hydrogen.

### Molecular docking

The structure with the lowest energy conformation was prepared for docking study with AutoDockTools1.5.6 and docked using Autodock4.2.5 [25]. The protein was prepared by removing the water molecules, adding polar hydrogen, and assignment of the Kollman united-atom partial charges. A grid box with dimension of 40×40×40 and 0.375Å spacing was created to allow the ligand to explore particular space in the macromolecules. Docking run employed Genetic Algorithm with a total of 100 docking cycles. Number of individuals in population was set at 150 while maximum number of energy evaluations and of generation were 2.5 Millions and 50,000, respectively. Rate of gene mutation and of crossover were 0.02 and 0.8, respectively. The ligands with highest binding affinity were visualized using Discovery Studio Visualizer4.1 [30]. As a confirmation, second molecular docking experiment was performed using Autodock Vina [45]. Validation was done with the output of 25 cycles of docking to inspect for consistency in the calculated binding energy. Consensus was made from best docking models, resulted in one model that was adopted for further analysis. Furthermore, the structure of CNL was also subjected onto molecular docking with its natural ligands to obtain view on the CNL - ligand interaction. The grid box dimension and experimental setting was kept similar to that of Autodock4.2.5 for comparable results.

### B-cell epitope region prediction

Recognition by the B-cell was evaluated *in silico* exploiting various programs [6, 8, 20, 21, 34, 38, 39]. The outputs of prediction program other than Ellipro were collected to establish a peptide library. The level of confidence for each prediction program (except for Ellipro) was maintained at minimum of 75–80% and the length of epitope was limited to a range of 5–20 amino acids. Screening was performed by alignment of peptides, in which peptides with overlap of more than three amino acids were kept. Peptides with the most overlap were regarded as hotspots, which are consensus of peptides from all prediction programs. This consensus was then consulted with the output of Ellipro. For Ellipro, only output scores better than 60% (0.6) were accounted for, in order to prevent excessive elimination of any possible B-cell epitope region. The output from Ellipro was made primary because its prediction is based on the protein structure.
